# Effects of Swimming Pool Conditions and Floor Types on White Roman Geese’s Physical Condition Scores and Behaviors in an Indoor Rearing System

**DOI:** 10.3390/ani12233273

**Published:** 2022-11-24

**Authors:** Shih-Chieh Liao, Pei-Xuan Lyu, Shih-Yi Shen, Chih-Chang Hsiao, Ching-Yi Lien, Sheng-Der Wang, Tsung-Yi Lin, Po-An Tu

**Affiliations:** 1 Changhua Animal Propagation Station, Livestock Research Institute, Council of Agriculture, Changhua 512, Taiwan; 2 Livestock Research Institute, Council of Agriculture, Tainan 71246, Taiwan; 3 Hsinchu Branch, Livestock Research Institute, Council of Agriculture, Miaoli 36848, Taiwan

**Keywords:** White Roman geese, indoor rearing, swimming pool, floor types, physical condition score, behaviors

## Abstract

**Simple Summary:**

In most areas, domestic geese are still raised in free-range, semi-intensive, or backyard farms owing to their high adaptability to various environments and excellent fiber digestion. In many Asian countries, waterfowl production is severely affected by ongoing avian influenza biosecurity concerns. Numerous intervention policies are being implemented in Taiwan to limit the use of free-range or outdoor-based poultry farming. In contrast to the free-range rearing system, indoor rearing involves higher housing construction costs and a more restrictive living environment. Among various facilities in goose houses, an open water source is a major factor affecting goose welfare. Because waterfowl are strongly water-oriented, they require water for dabbling, bathing, grooming, swimming, and certain reproductive behaviors. This study investigated the effects of swimming pool conditions and different floor types on the physical condition scores and behaviors of White Roman geese. Offering swimming pools and using perforated plastic floors improved physical condition scores and reduced feather damage in White Roman geese. Moreover, providing swimming pools decreased injurious pecking and diversified other behaviors.

**Abstract:**

Biosecurity problems, including the continual risk of avian influenza spread by wild birds, have severely affected traditional free-range waterfowl production systems. Regulations and techniques for indoor goose production require more considerations for animal welfare. This study investigated the effects of swimming pool conditions and different floor types on the physical condition scores and behaviors of indoor-reared White Roman geese. A total of 48 male and 48 female White Roman geese reared from the age of 15 to 84 days were randomly allocated to pens with or without a swimming pool and with either mud or perforated plastic floors. Providing a swimming pool improved geese’s eye and feather cleanliness and breast blister scores at the age of 84 days. Compared with geese reared on a mud floor, those reared on a perforated plastic floor had better feather cleanliness and higher breast blister scores at the age of 56 and 84 days. Providing a swimming pool to indoor-reared geese may reduce the proportion of abnormal behaviors, such as injurious feather pecking, by increasing water-related behaviors. This study suggests a more appropriate environment design for better balancing commercial goose production with animal welfare in an indoor rearing system.

## 1. Introduction

Global supply of goose meat was mainly produced in China, Egypt, Myanmar, Madagascar, and Ukraine in 2020, and China accounted for more than 95% of the goose meat produced in this year [1]. In Taiwan, goose is the third most commonly farmed poultry species produced after chicken and duck, with the White Roman goose being the main breed. Goose farming and consumption have been part of human culture for thousands of years. Geese are well adapted to diseases and cold conditions, require lower equipment investment in poultry houses, and have higher fiber digestibility than other poultry species [2,3]. Extensive (free range or agroforestry) and semi-intensive farming can reduce feed costs and provide goose meat rich in tocopherols, retinols, and polyunsaturated fatty acids [4,5]. The semi-intensive goose farming system would provide geese partial or full time access to outdoor sports fields with more activity space, allowing them to forage or providing them with additional roughage or fresh grass in comparison with intensive goose farming, which usually raises geese indoors with complete rations. Therefore, most geese are still raised in backyards, semi-intensive farms, and free-range or extensive farms in most regions of the world [2,6,7]. Before the avian influenza pandemic that occurred in 2014, most goose farms in Taiwan used semi-intensive farming and built swimming pools in outdoor sports fields with mud floors. However, this type of rearing has increasingly become a biosecurity risk because waterfowl can transmit diseases in commercial and natural open water systems [8]. The rapid spread of highly pathogenic H5 avian influenza through migratory birds since 2014 has resulted in numerous poultry deaths and culling and has thus led to substantial economic losses for poultry producers in various countries [9,10,11]. Taiwan is a part of the migration route of birds that migrate from Siberia and pass through China, Korea, and Japan on their way to reach Taiwan for winter. A goose farm’s open feed and pools provide a comfortable resting place for migratory or resident birds, attracting them to stop and forage in the farm. This phenomenon exposes geese to a substantially high infection risk. H5N2, H5N3, and H5N8 were among the most pathogenic types of avian influenza that caused numerous goose deaths in 2015 [12], reducing the relative output value of Taiwan’s goose industry by 64.2%. Taiwan’s goose industry lacks a vast hinterland and is more regionally concentrated compared with that in other goose-producing countries. Preventing the spread of avian influenza from one farm to another is difficult. To prevent contact between wild birds and geese, the government has implemented a stricter intervention policy since 2015. Thus, in Taiwan’s goose industry, the feeding method is changing, with an increase in the demand for indoor rearing.

An indoor rearing system is prone to animal welfare problems, causing diseases such as footpad dermatitis, injurious feather pecking, and breast blisters, which can affect the performance of poultry. Therefore, awareness regarding poultry animal welfare is increasing in many countries. An animal friendly environment can help breeders protect their animals from diseases and reduce their economic losses. A standard for animal welfare has been established by the Farm Animal Welfare Council in 1993 that specifies five freedoms for animals: freedom from hunger and thirst; freedom from discomfort; freedom from pain, injury, or disease; freedom to express normal behavior; and freedom from fear and distress. However, differences exist between animals and their normal behaviors. As waterfowl, geese have water-oriented living habits, and they require water to swim, bathe, and even breed to satisfy their physiological needs [13,14]. Domestic geese have inherited diverse behavioral patterns from their wild ancestors, and they spend considerable time performing complex preening behaviors [13,14]. Their preening procedures are elaborate and similar to those of ducks [14]. After bathing, geese perform various shaking, washing, snapping, and other actions to remove water and foreign objects from their bodies and arrange their feathers. Then, they exhibit oiling behavior, in which they apply oil from their uropygial gland above the tail to feathers; this behavior is crucial for waterproofing and thermal regulation [13]. The standard recommendations of the Standing Committee of the European Convention for the Protection of Farm Animals suggest that when raising geese indoors, open water sources should be provided as much as possible even in the absence of a pool [13]. Moreover, recent studies have demonstrated that water-related behaviors are irreplaceable for waterfowl [13,14,15,16,17]. Open water sources can enhance the well-being and behavior of waterfowl, such as ducks and geese. Furthermore, they can improve scoring indicators, such as feather quality; feather, eye, and nostril cleanliness; gait; and footpad dermatitis [14,15,16,17,18,19,20,21,22,23,24]. However, a water pool is a crucial medium through which ducks can transmit avian influenza virus [25,26,27]; when they drink, dabble, or preen in virus-contaminated pools, they may become infected, posing a high biosecurity threat to poultry farms.

Perforated floors are increasingly being used for intensive duck and goose farming [8,16,17,28,29,30,31,32,33]. By reducing the likelihood of birds being exposed to manure, dust, and pollutants, perforated floors maintain biosecurity more effectively than litter floors [32]. Moreover, compared with litter floors, perforated floors produce less harmful gases (carbon dioxide and ammonia) through the fermentation of organic matter, such as wet material, dripping material, and manure [16,34,35]. Furthermore, perforated floors reduce waste disposal costs after each feeding. The use of perforated floors in poultry production can maintain satisfactory eye, nostril, and feather cleanliness and improve immune and growth characteristics [16,28,29,30,32,34,35,36,37,38,39]. However, perforated floors may impede foraging behavior, thereby increasing the chance of feather pecking, affecting athletic performance, and causing thoracic injuries and footpad dermatitis [16]. Thus, the effect of the use of litter or perforated floors for goose production on animal welfare indicators should be investigated.

Given consumer preferences for free-range products, goose products generated in a free-range environment may be more popular [40]. The cost of building and operating goose houses indoors is higher for farmers who raise poultry outdoors. Moreover, indoor rearing is more rigid and subject to more restrictions than free-range rearing for facilitating management and improving productivity. Thus, in such cases, the welfare of geese is easily overlooked. Although geese reared indoors benefit from improved biosecurity, their welfare is compromised. Thus, strategies should be developed to maximize goose welfare while maintaining biosecurity and farmer benefits. In this study, we investigated the effects of swimming pools in goose houses and two-floor types on the welfare of goose through the physical condition score and behaviors of White Roman geese.

## 2. Materials and Methods

**Figure 1:**
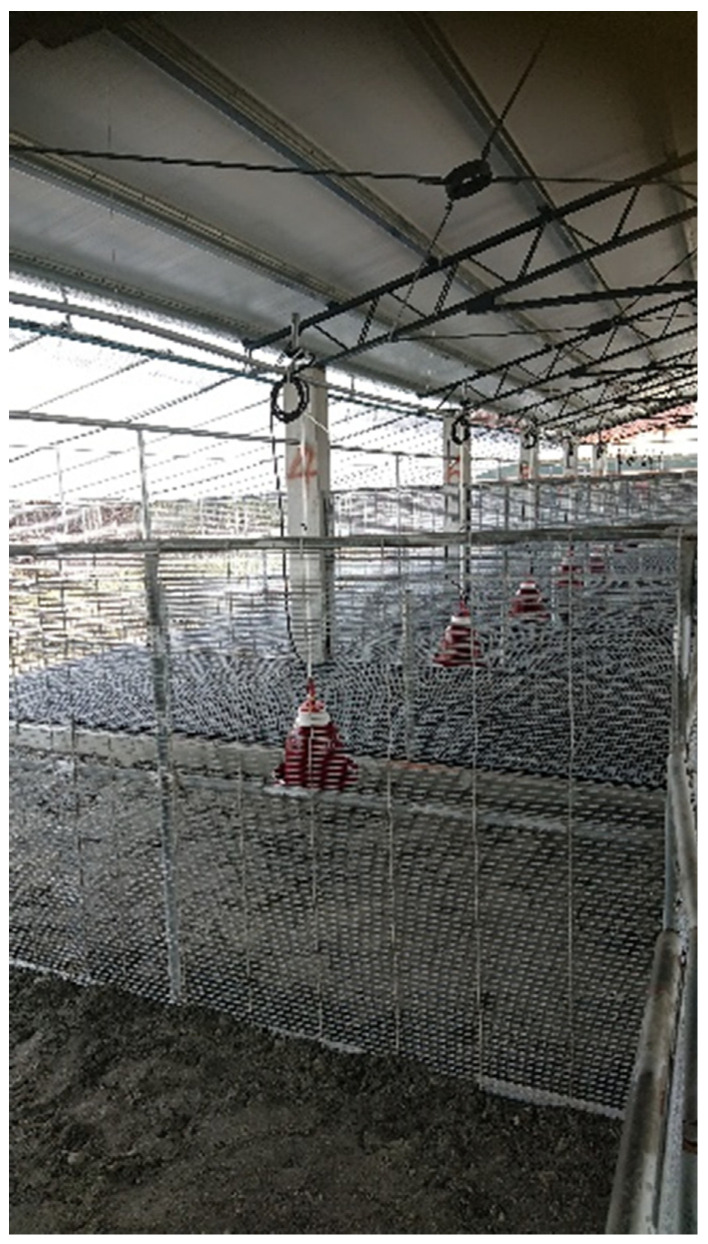
The indoor goose house set-up in this experiment. Water was provided by red narrow-lip bell drinkers. Pens were separated using wire meshes. A plastic greenhouse film was placed on top of the pool to provide natural light. The roof above the feeding and resting areas was also covered with polyurethane foam sandwich panels to prevent geese from interacting with wild birds.

**Figure 2:**
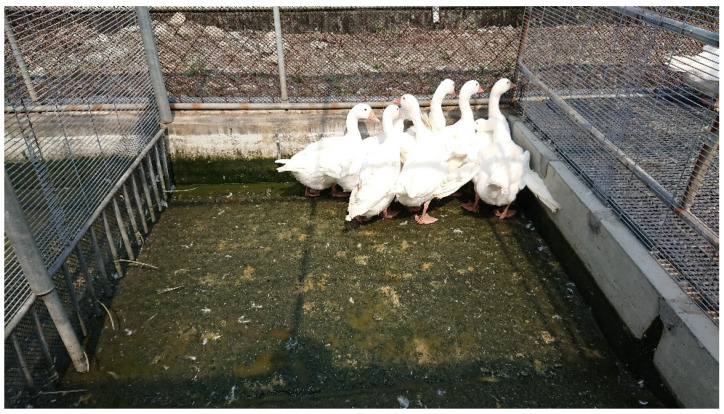
The swimming pools of the pens in this experiment. The plastic greenhouse film was used at the top of the swimming pool to provide natural light. The pool measured 2.57 m long and 2.10 m wide, respectively. Its deepest point is 38 cm, and at its entrance there is a gentle slope so that the geese can enter slowly.

In this study, White Roman geese were raised at Changhua Animal Propagation Station, Livestock Research Institute, Council of Agriculture, Executive Yuan, Taiwan. Animal experimental procedures were performed in accordance with Taiwan’s animal protection laws and were approved by the Institutional Animal Care and Use Committee of Changhua Animal Propagation Station (approval number: 10806). A detailed description of the management of geese at different stages has been provided by Liao et al. [24]. An indoor house was surrounded by plastic antibird nets to rear geese (Figure 1). A wire mesh was used to separate each pen. A 2.57 m × 2.10 m and 38-cm-deep swimming pool occupied 50% of the each pen’s space, and a plastic greenhouse film placed at the top of the swimming pool provided natural light. The geese were initially provided with 8 cm of water, which gradually increased to 38 cm at 42 days, and remained at that depth until the experiment ended (Figure 2). A polyurethane foam sandwich panel was used for the roof above the feeding and resting areas. This design was used to prevent contact between geese and wild birds in accordance with Taiwan’s regulations for indoor goose rearing.

### 2.1. Experimental Design

We estimated the required sample size by using the standard Type I error α = 0.05 and Type II error β = 0.5. On the basis of the average and standard deviation determined in our previous study, we calculated the standardized effect size (d = 0.4). The nondirectional null hypothesis was considered as the absence of any difference (Ho: μ1 = μ2) between the two-tailed distribution. Using G*Power software (version 3.1.9.7, Heinrich-Heine-Universität Düsseldorf, Düsseldorf, Germany) [41], we determined the required sample size as 84. We used 96 animals to detect a treatment difference. The geese were randomly assigned to a 2 × 2 factorial design with different swimming pool conditions and floor types. Each treatment consisted of three pens with eight geese in each. They were either offered a swimming pool (S) or not (N). The floor types were mud floors (M) or perforated plastic floors (P). We applied 8-cm-high clean earth layers and 30-cm-high perforated plastic to the existing cemented floor in the house for the treatment of M and P, respectively. For those who were not provided a water pool in N × M and N × P, perforated plastic floor and earth covered the water pool space, respectively.

### 2.2. Physical Condition Scoring

On the basis of studies conducted by O’Driscoll and Broom [20] and Karcher et al. [29], we developed a modified physical condition scoring system (Table 1). A visual inspection was performed at 28, 56, and 84 days of age to score each goose according to the standards listed in Table 1. Geesese were picked up and physically restrained by the staff, and then evaluated for physical condition scoring by another fixed training staff member between 09:00–12:00 in the morning. Scores were recorded at the pen level, and the average score was calculated for all geese in the pen. The number of geese at 28, 56, and 84 days of age affected by injurious pecking and the feather quality score (FQS) were recorded for each pen.

### 2.3. Behavior Observation

A modified standard based on that described by Baéza et al. [42] was used to observe time budgets associated with each goose’s behaviors and social interactions. At the age of 56 and 84 days, we measured each pen’s time budgets associated with behaviors and social interactions in the morning (9 to 12 AM) and afternoon (13 to 16 AM). In each time segment, three observations with a duration of 2 min were made for each pen. Various behaviors of geese were carefully recorded, including sleeping, standing, sitting, eating, drinking, walking, preening, pulling the feather of another goose, pecking at the environment, disturbing another goose, and swimming (Table 2).

### 2.4. Statistical Analysis

Eye and feather cleanliness, feather quality, and breast blister scores were not transformed before their analysis was performed using generalized linear mixed models [43]. In the models, pool conditions and floor types and their interaction were included as response variables. Mean values and standard errors of the mean (SE) were calculated for all parameters. Tukey’s post-hoc test was used for detecting the differences between the mean values. Behavioral observation variables with non-normal distribution were analyzed using the nonparametric Kruskall–Wallis and Mann–Whitney U tests. Differences in the occurrence of disorders, such as pecking and disturbing behaviors, were analyzed using the chi-square test. A *p* value of <0.05 indicated statistical significance.

## 3. Results

### 3.1. Physical Condition Scoring

**Figure 3:**
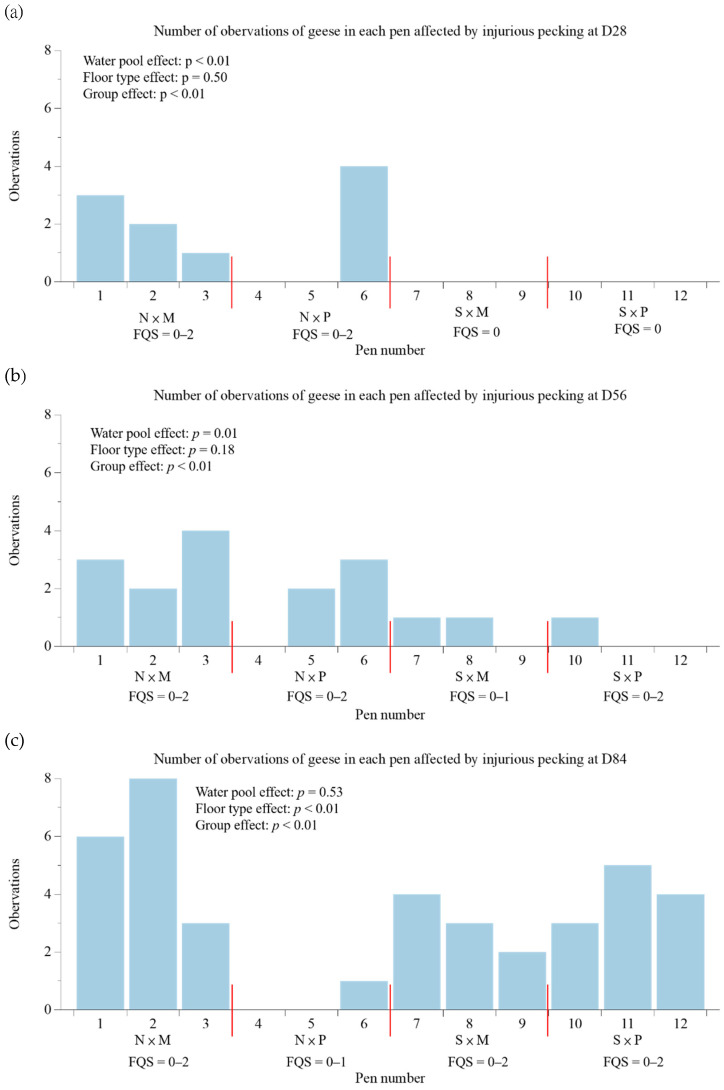
Effects of different swimming pool conditions and floor types on the number of geese affected by injurious pecking in each pen and the feather quality score (FQS) at the age of 28 (**a**), 56 (**b**), and 84 (**c**) days (n = 3). The number indicated under the X axis represents the pen number. N × M = no swimming pool and mud floor; N × P = no swimming pool and perforated plastic floor; S × M = with swimming pool and mud floor; S × P = with swimming pool and perforated plastic floor.

Table 3 presents the physical condition scores. At the age of 84 days, extremely significant interaction effects of the swimming pool conditions and floor types were observed for eye cleanliness, feather cleanliness, and breast blister scores (*p* < 0.01), while the interaction effect of feather quality showed a significant difference (*p* < 0.05). A similar pattern was observed for feather cleanliness and breast blister scores at the age of 56 days. The N × M group had higher feather cleanliness and breast blister scores at the age of 56 days, as well as eye and feather cleanliness and breast blister scores at the age of 84 days. The N × M group had significantly higher feather quality at the age of 84 days than the N × P group (*p* < 0.05). In addition, compared with the S group, the N group exhibited higher feather cleanliness, feather quality, and breast blister scores at the age of 56 days and eye and feather cleanliness and breast blister scores at the age of 84 days (*p* < 0.05). Compared with the M group, the P group had improved feather cleanliness and breast blister scores at the age of 56 days and eye and feather cleanliness and breast blister scores at the age of 84 days (*p* < 0.05). No difference in the nostril cleanliness score was observed between the groups. A higher proportion of geese in the N × M group were affected by injurious pecking at the age of 28 days than those in the S × M and S × P groups (*p* < 0.01), and at the age of 56 days than those in the S × P group (*p* < 0.01; Figure 3). At the age of 84 days, a lower proportion of geese in the N × P group were affected by injurious pecking (*p* < 0.01). At the age of both 28 and 56 days, a higher percentage of geese were affected by injurious pecking in the N group than in the S group (*p* < 0.01). In addition, at the age of 84 days, a higher proportion of geese were affected by injurious pecking in the M group than in the P group (*p* < 0.01).

### 3.2. Behaviors

Table 4 presents differences in the behaviors of geese at different ages based on the swimming pool conditions and floor types. Significant interactions of the swimming pool conditions and floor types were noted for sitting and swimming at the age of 56 and 84 days: preening, pulling the feather of another goose, and disturbing another goose at the age of 56 days; and pecking at the environment at the age of 84 days (*p* < 0.05). At the age of 56 days, the N × P group exhibited a higher percentage of sitting behaviors (*p* < 0.05), and the N × M group demonstrated a lower percentage of preening and a higher percentage of disturbing another goose than the S × M and S × P groups (*p* < 0.05). Moreover, the S × P group exhibited the lowest percentage of pulling the feather of another goose (*p* < 0.05), and the S × M and S × P groups demonstrated a higher percentage of swimming than the N × M and N × P groups (*p* < 0.05). At the age of 84 days, the N × M and N × P groups exhibited a higher percentage of sitting than the S × M group (*p* < 0.05), and the N × P group demonstrated a lower percentage of pecking at the environment than the S × M group (*p* < 0.05). In addition, the S × M and S × P groups exhibited a higher percentage of swimming than the N × M and N × P groups (*p* < 0.05). At the age of 56 days, the S group demonstrated higher percentages of drinking, preening, and swimming and lower percentages of pulling the feather of another goose and disturbing another goose than the N group (*p* < 0.05). Moreover, at the age of 84 days, the S group exhibited higher percentages of pecking at the environment and swimming and lower percentages of sleeping and sitting than the N group. Furthermore, at the age of 84 days, the M group displayed higher percentages of preening and pecking at the environment (*p* < 0.05).

## 4. Discussion

Raising poultry outdoors is risky because of the threat of a new type of avian influenza and the potential for irreparable economic losses. In many countries, such as Thailand and Taiwan, intervention policies have been introduced to limit free-range or outdoor rearing in poultry farms and strengthen control and restrictions on backyard farming [24,44,45]. Because of these policies, the poultry industry is forced to raise ducks and geese indoors to prevent their contact with wild birds; this has led to the increased focus on intensive goose farming. Intensive and extensive farming significantly differ in terms of the feeding environment, management, and diet composition, which affect goose market age, feed conversion rate, carcass quality, feather quality, and animal behaviors [3,14,40,46,47,48]. The cost of construction and equipment for intensive farming is high. For the convenience of management, the breeding environment is considerably restricted, which leads to an increase in stocking density and a substantial reduction in environmental diversity. This phenomenon not only affects goose growth, but also leads to many farm animal welfare problems, such as physical trauma, feather pecking, footpad dermatitis, breast blisters, and lameness [40,46]. Among various goose house facilities that may affect the well-being of geese, open water sources play a crucial role. Chen et al. [18] reported that 4-week-old goslings that received water baths had higher uropygial gland weight than those without access to open water. In ducks, a lack of access to a water pool may reduce their tail gland weight, relative weight, length, width, and height [23]. Frequent exposure to cold water stimulates the growth of goose feathers and protects their bodies [49]. Moreover, providing bathing facilities and rearing geese in extensive conditions promotes feather growth and down production [49]. Kozák [49] reported that geese reared under overcrowded conditions without bathing facilities exhibited delayed feather development. When geese are unable to express their food-seeking or investigative pecking behaviors in barren environments, they are likely to redirect their pecking behaviors toward other geese [13]. According to our results, the lack of swimming pools can increase the percentage of behavior of pulling the feather of another goose at 56 days of age in White Roman geese. Based on the research of ducks that feather pecking is associated with the absence of water pools [17,50,51], we speculate that this may be the result of monotonous environments causing abnormal behavior in geese. In addition to causing weakness in geese, feather pecking can affect the appearance and quality of feathers. Thus, the prevention of feather pecking in geese is crucial for not only animal welfare, but also economic reasons.

No animal-based measures are currently available to examine the welfare of geese on farms in commercial meat production systems [52]. Behavioral and appearance scoring systems for feather quality, body damage, feather contamination, hock damage, breast blisters, and footpad dermatitis are commonly used for examining animal welfare [14,16,17,46,48,52]. Our experimental results revealed that after geese raised in the house were provided with a pool, their eye cleanliness, feather quality, and blister scores improved significantly. This finding is similar to that reported by Farghly and Mahmoud [22] that providing ducks with 4 or 6 h of outdoor pool time per day improved their feather quality and breast blister scores. O’Driscoll and Broom [20] reported that Peking ducks raised in an environment with open water sources had improved feather and nostril hygiene and decreased nostril blockage compared with those raised with narrow-lip bell drinkers. The provision of small ponds, troughs, or overhead showers increased the proportion of ducks with clean eyes, nostrils, and feathers [19]. In addition, perforated plastic floors significantly improved the eye and feather cleanliness and blister scores of geese. Similarly, Karcher et al. [29] observed that plumage hygiene declined with age in ducks reared on the litter floor, but they noted better feather hygiene in ducks reared on a plastic slatted floor. The feather condition and gait scores of ducks reared in plastic nets were improved compared with those of ducks reared on sawdust bedding [36]. Compared with litter floors, perforated plastic floors improved feather cleanliness in broilers [34,35,38,39]. These results support the positive argument that the use of perforated floors can improve physical condition scores in poultry; birds raised on perforated floors have improved feather hygiene because of the prevention of their contact with manure and litter. However, Fraley et al. [30] observed that ducks raised on pine shaving litter had cleaner eyes, feathers, and nostrils than ducks raised on plastic slatted floors. Almeida et al. [35] indicated that broilers raised on perforated plastic floors under heat stress conditions may be more likely to develop breast lesions than those raised on wood shavings. Geese force-fed in individual crates for foie gras production easily developed pressure sores on the sternum [14]. The differences in findings may be attributable to variations in bird breed, age, weight, floor design, housing conditions (temperature, humidity, ventilation, and heat stress), and management practices (activity space, stocking density, and abnormally high humidity in the house caused by insufficient cooling) [16,17,31,53,54]. Our experiment revealed that compared with geese reared on a perforated plastic bed, those reared on a mud floor had significantly improved eye and feather cleanliness and breast blister scores after they were provided with a swimming pool and achieved the same scores as those reared on the perforated plastic bed. This finding indicates the importance of providing goose bathing facilities in the house environment to maintain the cleanliness and health of geese. Moreover, it suggested that when considering the aspect of maintaining goose feather cleanliness and wellbeing, pool provision should be given more priority than floor type.

Although we did not observe any effect of the floor type on the feather quality score of geese, the proportion of geese whose feather quality was damaged by injurious pecking decreased when geese were raised on perforated plastic floors at the age of 84 days. Lin et al. [33] reported that geese raised on a cement strip floor had longer main wing feathers at the age of 84 days than those raised on a cement floor. Chen et al. [36] observed that ducks raised in plastic nets had better feather quality scores. In this study, geese provided with swimming pools had higher feather quality scores at the age of 56 days; however, their scores at the age of 84 days were identical to those of geese without swimming pools. We found that the 84-day-old feather quality scores of the S × M and S × P groups were intermediate compared with those of the N × M and N × P groups. Analysis of the distribution of the feather quality scores of individual geese revealed that the proportion of feathers affected by injury pecking increased with the age of geese. Provision of a pool reduced injury pecking at 28 and 56 days, but no difference in scores was observed between those provided a pool and those not provided a pool at the age of 84 days. This may be because, with aging, geese spend more time dabbling in water to cool down in hot weather, causing them to repeat the act of preening continuously, which leads to a decline in their feather quality. Feather damage may result from grooming or injurious pecking in poultry [14,55,56]. Ducks could exhibit an auto-mutilation behavior known as self-directed feather-picking, which involves excessive removal of feathers during grooming [14]. Dong et al. [55] have described different types of injury pecking, including feather pecking, feather picking, cannibalism, and aggressive pecking. A study conducted in chickens indicated that feather-picking birds were more likely to practice preening behavior, and that preening oil might be a factor responsible for feather pecking [56]. Jones and Dawkins [53,54] demonstrated that older Peking ducks exhibited panting behavior caused by thermal stress at lower temperatures as well as more wet and dry preening with aging (from 23 to 43 days). Babington and Campbell [17] reported that providing open water sources during hotter seasons may be crucial for older ducks to promote thermoregulation and mitigate heat stress. Future studies should determine whether wet preening after dabbling in water to cool down causes excessive preening, which damages feather quality, in waterfowl.

Geese may respond differently to changes in their environment, with captive geese exhibiting higher stress levels than wild geese, but captive geese more favorably adapt to stress encountered in management systems [14]. Free-range geese exhibit lower levels of fearfulness, feather pecking, wing flapping or feather shaking, and preening, and higher levels of foraging, resting, or standing than geese reared intensively [48]. In enriched environments with pools, laying geese exhibited increased behaviors, including swimming, flapping, stretching, feathering, and oiling [57]. In our study, we observed that geese raised in houses with a swimming pool increased their drinking, preening, and swimming behaviors at the age of 56 days; reduced their feather pulling, disturbing other geese, sleeping, and sitting behaviors; and increased pecking in the environment and swimming at the age of 84 days. This finding indicates that goose behavior can be enhanced by providing a swimming pool. By spending more time on water-related activities, geese may be less likely to engage in abnormal behavior (such as feather pecking and disturbing others), sleeping, and sitting. Similarly, Mohammed et al. [51] reported that the absence of a water bath while rearing Muscovy ducks increased the incidence of feather pecking. Ducks displayed less aggressive pecking behavior in outdoor systems with outdoor pools than in indoor systems without pools [50]. Mi et al. [23] reported that the provision of water pools increased the proportion of preening behavior in Sanshui White ducks at the age of 2, 3, 4, 5, and 6 weeks.

In this study, geese raised on a mud floor exhibited an increased frequency of preening and pecking at the environment at the age of 84 days relative to those raised on a perforated plastic raised floor. According to the physical condition score results of this experiment, we speculate that the environment of geese in the N group became muddy over time because their bodies are more easily contaminated with dirt, causing them to perform more preening compared with geese in the P group. El-Edel et al. [50] suggested that ducks raised without a pool may experience contamination more frequently, which may explain the increase in their total body care behavior. Abdel-Hamid et al. [58] found that Muscovy ducks raised in deep litter exhibited a higher frequency and duration of foraging and pecking; a higher frequency of feather preening, head shaking, wing flapping, and body shaking; and a lower frequency and duration of water-related preening than those raised in cages. In his study, the caged group had higher stocking density than the deep litter group (5 duck/m^2^ vs. 4 duck/m^2^), whereas all treatments in our experiment had the same space size and density (0.74 goose/m^2^); this may be a reason for the partial discrepancy with our findings. Yangzhou geese reared on a plastic wire floor exhibited increased feather pecking and preening behaviors [31].

Perforated floor materials may interfere with normal duck foraging behaviors, causing more injurious pecking [16]. We observed a higher proportion of pecking at the environment in geese reared on a mud floor than in those reared on a perforated plastic floor, partially validating the theory. Foraging is a natural behavior of poultry that presents an opportunity for exploration and interest in the environment [13,14,16,17]. Animal welfare means reducing negative welfare while increasing positive welfare to provide positive physical and mental experiences such as interest, comfort, and pleasure [59,60]. Based on this opinion, geese reared on mud floor showed better positive welfare by acquiring more behavior of pecking at the environment. Moreover, Chen et al. [36] observed more injurious pecking in ducks reared on plastic net bedding than in those reared on sawdust bedding when they had access to water pools. However, we noted no difference in the behavior of pulling the feather of another goose between geese reared on perforated plastic and mud floors. Furthermore, at the age of 56 days, the S × P group exhibited a lower proportion of injurious pecking than the S × M group, which is not in agreement with the results reported by Chen et al. [36]. The discrepancy in the findings might be related to differences in animal species, water pool designs, water pen space ratios, litter materials, floor designs, and stocking density [16,17,31,50]. The stocking density in this study was lower than that in other studies using perforated floors (0.74 goose/m^2^ vs. 1.01 to 7.5 goose/m^2^) [31,33,61,62].

Geese reared in free-range environments have better opportunities for expressing their normal behavior than those raised in intensive production systems. Environment enrichment is the primary cause [16,48]. Boz et al. [48] reported that geese exhibited lower feather pecking and higher foraging behaviors when raised on a free-range field. They defined geese foraging behavior as pecking or scratching on the ground and consuming feed at the feeder and water at the drinker, which is similar to our definition of pecking. However, we also considered feeding and drinking behaviors in our study. Natural sunlight, vegetation, and terrain variations are more likely to improve geese foraging behaviors. Maintaining the appropriate intensity, wavelength, and variations of indoor light is more tedious than those of outdoor light. Geese farmers restricted to indoor rearing by law in Taiwan have reported frequent feather pecking problems, resulting in low feather quality. This problem can be partially resolved by providing fresh grass to geese. Behavioral trends did not differ consistently between the treatment groups at the age of 56 and 84 days in our study, which might be due to differences in physiological factors, environmental factors, and temperature and humidity.

## 5. Conclusions

Overall, we determined that providing swimming pools and using perforated plastic floors improved the physical condition scores of White Roman geese and reduced their feather damage. By offering simple swimming pools, injurious pecking can be reduced, and other behaviors can be diversified. Because of ongoing avian influenza biosecurity concerns, waterfowl production is severely affected in many Asian countries. Researchers must put more efforts into developing an indoor rearing system considering biosecurity, animal welfare, and production efficiency. More extensive evaluations of animal welfare and behavior are required for rearing geese in different types of facilities.

## Figures and Tables

**Table 1:** The criterion of the physical condition score in geese.

Condition	Score	Definition
Eyes cleanliness	0	Eyes clear, clean, and bright.
	1	Dirt or staining around the eye area or any evidence of wet eye ring.
	2	Inflamed eye lids, conjunctivitis, eyes sealed shut or blind.
Nostril cleanliness	0	Nostrils with clean and clear air passageways.
	1	Some blockage visible when viewed from the side.
	2	Nostrils entirely blocked on at least one side.
Feather cleanliness	0	Adhering manure or staining on down or feathers < 10% (not including breast part).
	1	Adhering manure or staining on down or feathers 10–50% (not including breast part).
	2	Adhering manure or staining on down or feathers > 50% (not including breast part).
Feather quality	0	Normal: good coverage of down or feathers without any bleeding and some evidence of down/feather picking or damaged area (as evident by short and stubby down/feathers) less than 1 cm^2^.
	1	Moderate: Some evidence of down/feather picking or damaged area (as evident by short and stubby down/feathers) between 1 to 10 cm^2^.
	2	Worst: severe feather picking (as evidenced by blood) or damaged areas (as evident by short and stubbly down/feathers) of greater than > 10 cm^2^.
Breast blister	0	Normal: good coverage and cleanness of breast down or feathers.
	1	Moderate: dirty or discolored of breast down or feathers and feathers still intact.
	2	Worst: lesion and the uncovered skin of breast or the skin showed visible ulcers, scabs, bleeding, or swelling.

Note. O’Driscoll and Broom [20] and Karcher et al. [29].

**Table 2:** Ethogram of recorded behaviors of geese.

Behavior	Description
Sleeping	The goose sits on the ground with its head fully resting on its body and both eyes fully closed.
Standing	The goose stands on two or one foot, and may perform other activities while standing, such as preening, flapping, or drinking.
Sitting	The goose sits on the ground. Its eyes are open in comparison to when it is sleeping, regardless of whether the head or neck is leaning on the body.
Eating	The goose pecks inside the feeder for feed.
Drinking	The goose pecks drinking water from the bell-shaped drinker, holding its head up as it drinks.
Walking	During standing, the goose moves from one position to another.
Preening	The goose uses its beak to groom its wings and body feathers.
Pulling the feather of another goose	The goose pecks or pulls another bird’s feathers of body.
Pecking at the environment	The geese peck or bit objects around them, such as the ground, fences, water lines, drinkers, or feeders.
Disturbing another goose	The goose pecks another goose’s head, mouth, or body as it runs or walks, causing the other goose to stop or change its behavior.
Swimming	The geese sit or stand in the pool for swimming, shaking, washing, snapping, dabbing, or bathing.

Note. Adapted from Baéza et al. [42].

**Table 3:** Effects of different swimming pool conditions and floor types on the physical condition score of White Roman geese.

Experimental Groups	Eyes Cleanliness			Nostril Cleanliness			Feather Cleanliness			Feather Quality			Breast Blister		
	28 d	56 d	84 d	28 d	56 d	84 d	28 d	56 d	84 d	28 d	56 d	84 d	28 d	56 d	84 d
Swimming pool															
N	0.00	0.00	0.37	0.00	0.00	0.00	0.00	0.72	1.03	0.29	0.50	0.58	0.00	0.47	0.62
S	0.00	0.00	0.00	0.00	0.00	0.00	0.00	0.12	0.10	0.00	0.08	0.62	0.00	0.12	0.25
Pooled SEM	-	-	0.05	-	-	0.00	0.00	0.09	0.10	0.09	0.09	0.14	-	0.06	0.07
	NS	NS	**	NS	NS	NS	NS	**	**	NS	*	NS	NS	*	*
Floor type															
M	0.00	0.00	0.37	0.00	0.00	0.01	0.00	0.85	0.91	0.16	0.33	0.79	0.00	0.60	0.87
P	0.00	0.00	0.00	0.00	0.00	0.00	0.00	0.00	0.22	0.12	0.25	0.41	0.00	0.00	0.00
Pooled SEM	-	-	0.05	-	-	0.00	0.00	0.09	0.10	0.09	0.09	0.14	-	0.06	0.07
	NS	NS	**	NS	NS	NS	NS	**	**	NS	*	NS	NS	*	*
Swimming pool × Floor type															
N × M	0.00	0.00	0.75 ^a^	0.00	0.00	0.00	0.01	1.46 ^a^	1.63 ^a^	0.33	0.58	1.13 ^a^	0.00	0.96 ^a^	1.25 ^a^
N × P	0.00	0.00	0.00 ^b^	0.00	0.00	0.00	0.00	0.00 ^b^	0.44 ^b^	0.25	0.42	0.04 ^b^	0.00	0.00 ^b^	0.00 ^b^
S × M	0.00	0.00	0.00 ^b^	0.00	0.00	0.00	0.00	0.25 ^b^	0.21 ^b^	0.00	0.08	0.46 ^ab^	0.00	0.25 ^b^	0.50 ^b^
S × P	0.00	0.00	0.00 ^b^	0.00	0.00	0.00	0.00	0.00 ^b^	0.00 ^b^	0.00	0.08	0.79 ^ab^	0.00	0.00 ^b^	0.00 ^b^
Pooled SEM	-	-	0.07	-	-	-	-	0.13	0.14	0.13	0.13	0.21	-	0.09	0.10
	NS	NS	**	NS	NS	NS	NS	**	**	NS	NS	*	NS	**	**

d = at the age of days; N = no swimming pool; S = with swimming pool; M = mud floor; P = perforated plastic floor; N × M = no swimming pool × mud floor; N × P = no swimming pool × perforated plastic floor; S × M = with swimming pool × mud floor; S × P = with swimming pool × perforated plastic floor; Pooled SEM = pooled standard error of the mean; NS = not significant at *p* > 0.05; * *p* < 0.05; ** *p* < 0.01; ^a,b^ Different superscript letters on the same column indicate statistical significance (*p* < 0.05).

**Table 4:** Effects of different swimming pool conditions and floor types on average time budgets associated with the behaviors and social interactions of White Roman geese at different ages, recorded over a total of three daily scans of 2 min each per pen (percentage of geese expressing each behavior).

Behaviors (%)	Groups ^1^				Swimming Pool Effect	Floor Type Effect	Group Effect
	N × M	N × P	S × M	S × P			
Time budget and social interactions at 56 d^2^							
Sleeping	2.77	0.00	0.00	1.38	0.56	0.56	0.08
Standing	72.91	53.67	70.13	68.05	0.28	0.31	0.26
Sitting	25.69 ^b^	57.83 ^a^	29.86 ^b^	28.47 ^b^	0.08	0.17	0.02
Eating	0.00	0.00	0.00	0.00	-	-	-
Drinking	0.00	1.38	2.77	6.25	0.03	0.42	0.15
Walking	35.41	48.90	44.44	27.77	0.52	0.53	0.11
Preening	9.72 ^c^	16.66 ^bc^	31.94 ^ab^	36.11 ^a^	<0.01	0.41	<0.01
Pulling the feather of another goose	9.02 ^a^	11.80 ^a^	6.94 ^a^	0.69 ^b^	0.02	0.27	0.01
Pecking at the environment	4.16	11.60	9.72	11.11	0.48	0.11	0.20
Disturbing another goose	9.02 ^a^	8.03 ^ab^	2.77 ^bc^	2.08 ^c^	<0.01	0.69	0.02
Swimming	0.00 ^b^	0.00 ^b^	10.41 ^a^	24.30 ^a^	<0.01	0.28	<0.01
Time budget and social interactions at 84 d							
Sleeping	40.97	52.57	12.50	27.77	0.01	0.38	0.08
Standing	50.00	30.15	66.66	57.63	0.06	0.17	0.15
Sitting	53.47 ^a^	50.69 ^a^	11.11 ^b^	36.11 ^ab^	<0.01	0.72	0.02
Eating	0.69	0.00	1.38	1.38	0.48	0.58	0.77
Drinking	6.94	7.14	5.55	2.08	0.39	0.46	0.61
Walking	41.66	15.27	40.27	34.02	0.18	0.13	0.11
Preening	27.08	15.27	34.72	20.83	0.41	0.02	0.14
Pulling the feather of another goose	1.38	1.48	0.69	3.47	0.68	0.15	0.32
Pecking at the environment	7.63 ^ab^	2.18 ^b^	13.19 ^a^	8.33 ^ab^	0.01	0.03	0.01
Disturbing another goose	1.38	0.00	0.69	1.38	0.62	0.62	0.44
Swimming	0.00 ^b^	0.00 ^b^	22.22 ^a^	26.38 ^a^	<0.01	0.68	<0.01

^1^ Groups include N × M = no swimming pool and mud floor; N × P = no swimming pool and perforated plastic floor; S × M = with swimming pool and mud floor; S × P = with swimming pool and perforated plastic floor; ^2^ d = at the age of days; ^a,b,c^ Different superscript letters on the same row indicate statistical significance (*p* < 0.05).

## Data Availability

Data will be available from the first author upon request. The data are not publicly available due to the institutional policy.
